# Fifty Years With a Brooke Ileostomy: An Autobiographical Case Report

**DOI:** 10.7759/cureus.16980

**Published:** 2021-08-07

**Authors:** N. Marcus Thygeson

**Affiliations:** 1 Population Health, Adaptive Health, San Rafael, USA

**Keywords:** ileostomy, crohn’s disease, irritant dermatitis, obstipation, food blockage, viral gastroenteritis, gallstones, fat malabsorption, diet, nutrition

## Abstract

Permanent Brooke ileostomy continues to be a treatment option for selected patients with inflammatory bowel disease and cancer. This case report describes the author’s 50-year experience living with Crohn’s disease and a Brooke ileostomy, including the psychosocial and dietary adaptations required and the management of common complications such as peri-stomal irritant dermatitis, food blockage, and acute infectious diarrhea. Cross-sectional studies indicate that the quality of life with an ileostomy is usually good, but adaptation to an ileostomy is a life-long process.

## Introduction

Pan-proctocolectomy with a Brooke ileostomy was the standard surgical treatment for medically refractory inflammatory pan-colitis until the late 1980s [1]. Permanent end ileostomy remains a viable option for patients with ulcerative colitis and the treatment of choice for Crohn’s patients requiring pan-proctocolectomy [2].

Quality of life with a Brooke ileostomy is generally good [3-6] and comparable to that experienced with a normally functioning ileal pouch-anal anastomosis [7-8] in the absence of stoma-related morbidities such as stenosis, prolapse, and parastomal hernia [9]. However, the medical literature regarding life with an ileostomy is sparse and cross-sectional, longitudinal case studies are rare if not non-existent, and the median post-ileostomy follow-up is 15 years or less [3-9].

This case report describes the author’s experience of living with an ileostomy, including complications, adaptations, and impact on lifestyle and quality of life, for 50 years. My goal in writing this autobiographical case report is to provide a resource for my colleagues regarding the management of clinical phenomena specifically associated with the Brooke ileostomy and to describe how these phenomena evolve over time.

## Case presentation

I was diagnosed with Crohn’s ileocolitis in 1970, at the age of 17, and treated with sulfasalazine and prednisone. Shortly thereafter I developed acute peritonitis from a perforated terminal ileum and underwent an emergency laparotomy, resection of roughly 100 cm of the distal ileum, and creation of a Brooke ileostomy and mucous fistula, both positioned in the right lower quadrant. During the subsequent one-month hospitalization, I was taught by an ostomy nurse how to manage my two stomas and their associated appliances. I was told to expect a high ileostomy output that would decrease over time and to avoid popcorn and other high-fiber foods.

Over the next eight years, I continued to take sulfasalazine and low-dose prednisone because of low-grade symptoms that in retrospect may have been due to unrecognized diversion colitis. In 1978, mid-way through medical school, I decided to undergo a pan-proctocolectomy and removal of the mucous fistula, with the thought that this would minimize my Crohn's-related morbidity going forward. The prednisone and sulfasalazine were discontinued after the surgery. I have not required any further treatment for Crohn’s disease.

Peri-stomal skin irritation

Ileostomy effluent irritates the skin, causing peri-stomal irritant dermatitis with varying amounts of itching, burning, and pain. I have had a fluctuating level of peri-stomal skin irritation since my stoma was created. When mild, irritant dermatitis manifests as a ring or focal areas of erythema within 5 millimeters of the ileo-cutaneous junction. If not addressed timely, this can progress to shallow erosions or ulceration associated with a white pseudo-membrane. On one occasion, due to the use of a contaminated washcloth, I developed a peri-stomal yeast infection that responded to topical Mycostatin powder.

Minimizing contact between ileostomy effluent and the peri-stomal skin is key to preventing and managing peri-stomal skin irritation. Over the years, I have developed a satisfactory regimen for doing this, such that I am bothered by skin-irritation symptoms only a few days each month. One key to success is having a good stoma. My stoma is well-everted, with a circular ileo-cutaneous junction and a centrally positioned orifice.

A well-fitting appliance is essential. My first appliance was multi-part and required elaborate preparation, including the application of a separate Karaya skin barrier around the stoma. I would carefully wash and dry the skin around the stoma before attaching the new appliance, all the while hoping my high-volume ileostomy would remain “inactive” and not force me to repeat my skin prep or soil the new appliance as I applied it. With luck, I could complete the process in 30 minutes and then go three days before skin irritation required an appliance change. I later switched to a one-piece drainable stoma bag with paper adhesive and a Karaya skin barrier. This was much quicker to replace, although not more long-lasting. I now use a one-piece Hollister appliance with a convex faceplate and a “flextend” skin barrier/adhesive. My skin preparation regimen has become quite minimalist - a quick but careful wash with hot tap water and a clean washcloth. I change my ileostomy appliance in just a few minutes - usually in the morning before breakfast when the effluent is minimal - but the process is so quick, I can do it almost anytime (and anywhere).

Changing the appliance before, or as soon as possible after, skin irritation develops is also important. At first, I tried going five days before changing my current appliance, but often, I would feel some peri-stomal irritation during the last 24 hours. I now routinely change the appliance every four days and usually have little or no irritation around the stoma. In the past, I was loath to change the appliance any more often than “necessary,” mainly because it was inconvenient; but the inconvenience is minimal with the current appliance and prevention is better than cure. 

It is also essential that the appliance is well-centered on the stoma and firmly adherent to the peri-stomal skin. At my age, the skin just cephalad to the stoma tends to sag down onto the stoma when I’m standing up. Digitally retracting this skin as I place the appliance over the stoma enables me to achieve a good, circumferential seal with the adhesive. I also gently evert the adhesive with my thumbs just before applying the appliance. This creates a larger orifice so that I can slip it over the stoma without touching the mucosa of the stoma. As the appliance settles on the skin, the everted adhesive edges return to their normal narrower circumference, fitting snugly onto the skin immediately adjacent to the stoma.

Finally, as noted above, avoiding excessively liquid ileostomy effluent helps prevent skin irritation. My skin irritation has been much less of a problem since adopting a low-fat diet, as described below. I avoid rich, creamy sauces and halvah, of all things, for the same reason.

Nutritional issues

In 1977, both fat and vitamin B12 malabsorption were formally diagnosed, and I treated myself with medium-chain triglycerides (briefly) and monthly subcutaneous injections of cyanocobalamin. Some years later, I switched from injections to oral B12 and have maintained a B12 level in the normal range of 1000 mcg per day. I check my B12 level once a year.

The primary manifestation of fat malabsorption was high-volume, watery ileostomy effluent after ingestion of fatty foods, especially animal fat. I chose to live with this, rather than give up favored foods until I belatedly realized that my peri-stomal skin irritation was aggravated by the fat-induced ileostomy effluent. A pescatarian Mediterranean diet has substantially reduced the watery post-prandial ileostomy output.

Vitamin D deficiency (25-hydroxy vitamin D < 20 ng/mL) was diagnosed in 2010. Since that time, I have needed daily doses of cholecalciferol 125 mcg (5000 IU) to maintain a vitamin D level greater than 30 ng/mL.

Diet-related obstipation and food blockages

Over the years, I have had multiple episodes of food-induced obstipation, resulting in at least two hospitalizations for intravenous fluids and observation. These episodes are induced by eating moderate to large amounts of roughage, such as raw carrots, broccoli, apples, cabbage, or pickles, unmixed with other, low-fiber foods. The symptoms associated with these episodes follow a “small bowel obstruction” pattern. First, there is a cessation of all ileostomy output, rather than the expected post-prandial increase in output, followed within a few hours by crampy mid-abdominal pain of varying intensity, often lasting for hours. In my case, nausea and vomiting have not occurred. Eventually, some fibrous material will pass from the ileostomy, followed by watery, foul-smelling effluent and diminishing cramps over the next 24-72 hours (depending on the severity of the episode), after which the ileostomy output returns to normal.

I have tried several approaches to managing these episodes of obstipation. Prevention is key, so I quickly learned to avoid eating large amounts of roughage at any given time, especially on an empty stomach. I limited myself to no more than half an apple, for example, and took care to chew it thoroughly before swallowing. During an episode, I found that drinking black tea seemed to help. As soon as I recognize an episode is occurring, I drink several cups of black tea (my patients with ileostomies have also found this helpful). More recently, I discovered that pressure on and massage of my right lower quadrant abdominal wall, just below the ileostomy appliance, regularly enables the passage of the offending material over the next hour and relieves the blockage. Probing the stoma with my little finger to beyond the abdominal wall fascia has in my case not been helpful.

In the past, these episodes occurred about once a year, but they have become much less frequent over time, as well as briefer and more manageable. I have increased my intake of high-fiber foods, although I am still careful about this.

Gallstones

In 1992, I experienced several episodes of post-prandial upper abdominal pain characteristic of biliary colic, and an ultrasound showed my gallbladder to be well-packed with calcified stones. I started taking ursodiol, 300 mg b.i.d., and had no further episodes of colic. Since discontinuing the ursodiol circa 2010, the biliary colic has not recurred although the gallstones persist.

Susceptibility to viral gastroenteritis

I have had several episodes of a norovirus-like illness, one of which required hospitalization. These episodes begin with the sudden, painless passage of a completely watery ileostomy effluent that fills the bag multiple times within a few hours. There may be associated nausea, vomiting, myalgias, or malaise. My first episode required hospitalization for fluid replacement. As would be expected with norovirus, symptoms abate after 24-48 hours. I am now extremely careful to avoid contact with anyone who has, or might have been exposed to, viral gastroenteritis. Over-the-counter anti-diarrheal medications and oral rehydration solutions may reduce the need for intravenous fluids.

Impact on physical and social activity

Since recovering from my initial illness and surgery, I have not experienced any physical limitations with respect to my activity. In my youth, I engaged in vigorous non-contact sports like touch football, softball, jogging, and squash. For many years, I avoided swimming because it loosened my appliance, but with my current appliance, I can swim with impunity. I wear a rash guard top to help other swimmers feel more comfortable when I am in the water with them.

Initially, I was quite bashful about my ileostomy. I never took my shirt off in public and was careful to wear clothes that made my ileostomy less noticeable. Despite that, co-workers occasionally ask me “what’s that bulge under your shirt?” A brief, basic answer to this question suffices to minimize our mutual embarrassment. Over time, I have become less shy about my ileostomy. In my forties, I started going to the gym, including using the locker room and showering. I found it psychologically liberating to do so. It seems natural at first to believe that one cannot do some (or even a lot of) things because of an ileostomy. I gradually became aware that I was setting these limits on myself and resolved to stop doing so.

I was a virgin at the time of my first surgery. At least briefly, I wondered if I was fated to stay that way. I soon learned, however, that the women I liked and who liked me accepted my condition. This even included dealing with the occasional nocturnal ileostomy leakage or frank “incontinence,” which can occur when the bag becomes too full and the seal fails, or the drainable spout comes undone. Over the years, these events have become rarer as appliances have improved. No doubt, the ileostomy curtailed my appetite for casual sex (a good thing, in retrospect), but having an ileostomy has not limited me sexually in any other way.

My quality of life is totally dependent on being able to obtain a sufficient supply of ileostomy appliances. To mitigate this risk and guard against disruptions of the supply chain, I have over the years been able to accumulate a stockpile of appliances sufficient to last several years. When traveling, I am always careful to pack at least twice as many ostomy bags as I expect to need. I divide them between my luggage items in case of loss or theft, so as to always have enough.

## Discussion

This case history describes many of the ileostomy-associated challenges identified in the literature, including intermittent skin irritation, occasional food blockages, susceptibility to gastroenteritis-induced hypovolemia, and the psychological and social adaptation to having an ostomy. Thirty years ago, Pemberton found that 93% of people with an ileostomy were satisfied overall with their quality of life, and 98% had returned to work or school [7]. Given improvements in ostomy appliances since that time, outcomes might be even better today. But more recent studies reveal that concerns about sexual function, leakage, and skin irritation are common, as are ileostomy-related changes in dress and diet [4]. Older patients may have a better quality of life [3].

The incidence and prevalence of food blockages is unknown, but they appear to be common, given that online educational materials for ostomy patients routinely address this topic. In addition to pressure and massage around the stoma, stoma intubation and lavage may be helpful for the relief of food blockages that do not resolve spontaneously. These methods are described in an online resource available from the United Ostomy Associations of America website (UOAA: How to treat ileostomy blockage. (2020) Accessed June 28, 2021: https://www.ostomy.org/wp-content/uploads/2020/10/Ileostomy_Blockage_2020.pdf). I hypothesize there is a kink in my ileum proximal to the ileostomy, that the bolus of roughage gets caught proximal to this kink and aggravates it, and that counter-pressure on the abdominal wall reduces the kink and allows the roughage to pass. It is also possible that there may be a residual stricture of the ileum proximal to the stoma. The decreasing frequency of these episodes over time, in my case, is notable and unexplained. I doubt it is due to changes in my diet; some foods that used to invariably give me trouble I can now eat with impunity. Perhaps it is due to changes in my gut microbiome, the disposition of postoperative adhesions, or amelioration of residual, Crohn’s-related, luminal narrowing. Patients with frequent episodes of food blockage may benefit from abdominal MRI to look for a significant adhesion or recurrent Crohn's disease.

I also developed gallstones and B12 and fat malabsorption, aggravated by the resection of a substantial segment of my terminal ileum. I have managed the fat malabsorption by limiting my fat intake and treated my biliary colic with ursodiol, a well-established though unusual treatment for symptomatic gallstones [10]. There are still many ileostomy complications I hope not to experience, including peri-stomal pyoderma gangrenosum [11], recurrent Crohn’s disease of the stoma [12], adenocarcinoma of the stoma [13], and squamous carcinoma of the ileo-cutaneous junction [14].

Other than the training I received immediately post-surgery, I learned how to manage my ileostomy on my own. The challenges I faced were never severe enough to motivate my seeking a consultation. I knew about the ostomy association but found my early interactions with other ostomates off-putting. I wanted to rise above my situation, not dwell on it. In retrospect, I might have benefited from more engagement with the broader ostomy community or additional consultation with an ostomy nurse. I might have learned earlier, for instance, about using counter-pressure below the stoma for relieving food blockages (UOAA, op. cit.).

At the time, the high-level dietary advice I received immediately after my ileostomy surgery seemed sufficient, but in retrospect, I would have benefited from more guidance. Recent studies from New Zealand, the United Kingdom, and Ireland suggest that inadequate dietary counseling remains an issue for many people with an ileostomy [3,15]. A recent evidence review summarizes the literature regarding the influence of dietary factors on ileostomy function and outcomes [16]. Most of the literature is based on expert opinion, not research. Approximately half of the people with an ileostomy report diet-related issues with their stoma. The most common food categories related to ileostomy function are fats, fiber, and alcohol. The authors conclude that “high-quality research investigating the effect of the dietary strategies.…on commonly associated outcomes relating to ileostomy management is needed to improve evidence-based advice.”

In my experience, physicians are often not well informed about ostomy management. My gastroenterologist colleagues tended to consult me about their patients’ ileostomy problems. Perhaps my “n-of-one” experience qualified me as an expert. One motivation for this case report is to improve ileostomy care by sharing my experience more broadly. Fortunately, there are now knowledgeable ostomy nurses in many if not most communities.

A limitation of this case report is that I have only experienced my ileostomy as a man. The literature is mixed with respect to how ileostomy-related quality of life varies by gender. Some report ostomy-related quality of life is worse for women than men [17]; others find no difference [3]. Intimacy is a major concern for both men and women with an ileostomy [18]. The experience of being pregnant with an ileostomy has been studied in a small case series. The involvement of an ostomy nurse may reduce uncertainty and increase the confidence of pregnant mothers with an ileostomy [19]. Rarely, the ileostomy may be obstructed by the gravid uterus [20].

Over time, my ileostomy has had a progressively less negative effect on my daily activities and quality of life. Skin irritation, food blockages, episodes of “incontinence,” and ostomy-related bashfulness have all gradually become less burdensome. Some of this progress is related to improvements in ostomy appliances but much is due to my slowly learning about how best to manage my condition.

Looking to the future, my quality of life depends on my continued ability to manage my stoma and ostomy appliance. I find it hard to imagine being dependent on someone else to do this for me. Loss of vision or function in even one of my upper extremities could be very debilitating. These were not things I thought about much when I decided to make my ileostomy permanent in 1978. My priority at that time was to maximize my physical ability to undertake the rigors of medical training, and I was concerned that an ileo-colic anastomosis would reactivate my disease. Removing my diseased bowel seemed to give me the best chance of being disease- and medication-free in the future. Neither my gastroenterologist nor surgeon argued against this, as I recall. In retrospect, I question our judgment, but I do not regret my choice. Fifty years later, I continue to live a full and rewarding life with my artificial, externalized “rectum.”

## Conclusions

The quality of life with a Brooke ileostomy is generally good, assuming the stoma is properly constructed. Problems like peri-stomal skin irritation, food blockages, and concerns about intimacy are common but manageable and tend to improve over time as the patient adapts to their ostomy. Most patients adjust quickly to having an ileostomy, but mastery may be a lifelong process. Consultation with ostomy nurse specialists, dieticians, and fellow ostomates should be encouraged.
